# A patient-centered framework for health systems engineering in gastroenterology: improving inpatient colonoscopy bowel preparation

**DOI:** 10.1186/s12876-021-01661-4

**Published:** 2021-02-27

**Authors:** Alexandra T. Strauss, Jennifer Yeh, Diego A. Martinez, Gayane Yenokyan, Janet Yoder, Ravi Nehra, Tara Feller, Kathy Bull-Henry, Ellen Stein, Lawrence C. H. Hsu, Haitham Al-Grain, Candice Zabko, Christopher Fain

**Affiliations:** 1 Division of Gastroenterology and Hepatology, Johns Hopkins University School of Medicine, 600 N Wolfe St. Blalock 465, Baltimore, MD 21205 USA; 2 Department of Internal Medicine, Johns Hopkins University School of Medicine, 601 N Caroline St, Baltimore, MD 21287 USA; 3 Department of Emergency Medicine, Johns Hopkins University School of Medicine, 601 N Caroline St, Baltimore, MD 21287 USA; 4 Johns Hopkins Biostatistics Center, Johns Hopkins University Bloomberg School of Public Health, 615 N Wolfe St, Baltimore, MD 21205 USA; 5 Department of Medical Nursing, Johns Hopkins University, 601 N Caroline St, Baltimore, MD 21287 USA; 6 Department of Pharmacy, Johns Hopkins Hospital, 601 N Caroline St, Baltimore, MD 21287 USA; 7 Operations Integration, Johns Hopkins Health System, 601 N Caroline St, Baltimore, MD 21287 USA; 8 Department of Anesthesiology, Johns Hopkins University School of Medicine, 601 N Caroline St, Baltimore, MD 21287 USA

**Keywords:** Colonoscopy, Bowel preparation, Quality improvement, Health systems engineering

## Abstract

**Background:**

Inpatient colonoscopy bowel preparation (ICBP) is frequently inadequate and can lead to adverse events, delayed or repeated procedures, and negative patient outcomes. Guidelines to overcome the complex factors in this setting are not well established. Our aims were to use health systems engineering principles to comprehensively evaluate the ICBP process, create an ICBP protocol, increase adequate ICBP, and decrease length of stay. Our goal was to provide adaptable tools for other institutions and procedural specialties.

**Methods:**

Patients admitted to our tertiary care academic hospital that underwent inpatient colonoscopy between July 3, 2017 to June 8, 2018 were included. Our multi-disciplinary team created a protocol employing health systems engineering techniques (i.e., process mapping, cause-effect diagrams, and plan-do-study-act cycles). We collected demographic and colonoscopy data. Our outcome measures were adequate preparation and length of stay. We compared pre-intervention (120 ICBP) vs. post-intervention (129 ICBP) outcomes using generalized linear regression models. Our new ICBP protocol included: split-dose 6-L polyethylene glycol-electrolyte solution, a gastroenterology electronic note template, and an education plan for patients, nurses, and physicians.

**Results:**

The percent of adequate ICBPs significantly increased with the intervention from 61% pre-intervention to 74% post-intervention (adjusted odds ratio of 1.87, *p *value = 0.023). The median length of stay decreased by approximately 25%, from 4 days pre-intervention to 3 days post-intervention (*p *value  = 0.11).

**Conclusions:**

By addressing issues at patient, provider, and system levels with health systems engineering principles, we addressed patient safety and quality of care provided by improving rates of adequate ICBP.

**Supplementary information:**

The online version contains supplementary material available at 10.1186/s12876-021-01661-4.

## Background

Effective colonoscopy is dependent on adequate bowel preparation by consuming a bowel cleansing medication prior to the procedure to allow for visualization of the colonic mucosa. Since colonoscopies are performed to evaluate for cancer, bleeding vessels, and other mucosal or structural abnormalities, visualization is paramount. A common problem is when hospitalized patients undergo bowel preparation for an inpatient colonoscopy, they frequently have an aborted procedure because the bowel preparation has failed (i.e., solid stool). This results in repeat bowel preparation, repeat or delayed procedures, compromised patient experience, and inefficient use of resources in the endoscopy unit.

Bowel preparation can be compromised by many factors [1, 2] including lower socioeconomic status [3], non-split dose bowel preparation, advanced age [4], male sex[5], unmarried status [6], narcotic use [2, 3, 7, 8], laxatives[3, 8], tricyclic antidepressants [5, 7, 9], diabetes mellitus [8], chronic constipation [8, 9], cirrhosis [5], stroke, and dementia [5]. Bowel preparation can also be compromised by poor compliance [5, 9, 10] and intolerance of preparation, such as vomiting [9]. Many of this prior work has focused on the outpatient setting as opposed to the inpatient environment. Hospitalized patients have additional risk factors that impact inpatient colonoscopy bowel preparations (ICBP). Studies have demonstrated ICBP rated as inadequate in about 65% of patients [2, 11]. The inpatient risk factors include acute illness, urgent indication for procedure, decreased level of mobility, timing, and coordination with other treatments and procedures [2, 5, 6, 10]. Furthermore, inpatient status has been shown to be an independent predictor of inadequate ICBP [5, 12–14]. The consequences of an inadequate ICBP include repeat procedures, increased length of stay (LOS) [2], and increased costs [3, 12].

Targeted interventions to improve ICBP have demonstrated success in specific situations to address patient and provider level factors. Such interventions include nursing education [15, 16], electronic nursing stool assessments [17], split-dose bowel preparation [17, 18], patient education [11, 19], and standardized order sets [17, 18, 20]. However, a knowledge gap exists for evidence-based guidelines since prior studies that have evaluated complex strategies for a good ICBP have not provided tools or comprehensive protocols as part of the publication to facilitate implementation elsewhere [17].

Health Systems Engineering (HSE) methods and tools (e.g., tracers, process mapping, cause-effect diagrams) are designed with the patient in mind to assess current complex systems, to improve processes (e.g., statistical process control charts), and to minimize variability (e.g., Lean process improvement) [21–23]. HSE principles have addressed a wide range of healthcare related problems successfully [24–26]. We hypothesize that using HSE principles and a multi-disciplinary team to perform a comprehensive analysis of the ICBP process can identify barriers and sustainable solutions. Our primary aim was to increase the absolute percentage of adequate and timely ICBP for our institution’s hospitalized patients by 10% within 6 months. Although a modest goal, it will form a foundation for continued improvement. Further, we believe the effects of a 10% improvement will be meaningful for both patients and providers. Our secondary objectives were to reduce LOS associated with the ICBP process. Our overall goal for this manuscript is to provide a detailed and transparent presentation of the HSE tools used for problem analysis that hospitalists, gastroenterologists, and other proceduralists can adapt to their institutions.

## Methods

### Context

In this quality improvement (QI) project at an academic tertiary care center, recognizing the complexity of intervening across multiple systems, such as primary care teams, gastroenterology consulting services, and the endoscopy unit, is important. Typically, providers have individual ICBP preferences, so understanding the culture regarding openness to change is important for a successful intervention.

Our sample included all patients that were hospitalized before and after the intervention that met the inclusion and exclusion criteria. Inclusion criteria were patients 18-years-old or older who had a non-emergent (i.e., non-critical care patients) inpatient colonoscopy. We included patients who underwent a colonoscopy in the 15-week pre-intervention period of July 3, 2017 to October 13, 2017 and the 15-week post-intervention period of February 26, 2018 to June 8, 2018. The period between groups was for intervention development and deployment. Exclusion criteria were patients in the intensive care unit, colonic foreign body, or colonic pseudo-obstruction. Our QI project was exempt by IRB and followed SQUIRE guidelines [27]. The main ethical consideration was that there may be specific patient situations or concerns that require flexibility for providers to use clinical judgement to make adjustments to the protocol when necessary. This study was otherwise minimal ethical risk to patients.

### Intervention

#### Phase I: Comprehensive Analysis of ICBP Process to Identify Barriers

We identified key stakeholders in the ICBP process and formed a multi-disciplinary team: residents, fellows, gastroenterologists, floor and endoscopy nurses, anesthesiologists, and pharmacists. The team defined the current ICBP process using HSE methods. One method was performing a tracer which is when team members observe the process from the initial step (i.e., decision to perform colonoscopy) through each additional step (i.e., nurse acts on order). Other HSE methods included creating a process map and cause-effect diagram. A process map involves the team drawing each step of the process including by not limited to patient movement and communication of information between providers. The team analyzed the process map for efficiency and safety. A cause-effect diagram is also created by the team and identifies all possible mechanisms leading to the outcome and categorizes them (e.g., supply problem, communication issue).

#### Phase 2: Solutions & Intervention Development

After reviewing the findings from Phase 1, subsequent solutions were then systematically brainstormed among our team to develop an intervention. The team identified that the high variability seen throughout the process led to many of the causes for a poor ICBP, so we decided a standardized protocol would be the necessary intervention. We designed arms of the protocol to address issues at the various levels of providers. Selection of aspects of the protocol were based on the quality of evidence in the literature and prioritization based on local subject matter expert experience. Operational practicalities were also considered. For example, we focused on changes our division could implement first as this is where we had the most control as opposed to infringing on operations outside of gastroenterology (i.e., scheduling procedure times). A preliminary ICBP protocol was piloted on the inpatient gastroenterology (GI) unit. The protocol went through multiple Plan-Do-Study-Act (PDSA) cycles (including additional tracers/observations, editing the process map, etc.) on the GI unit and again hospital-wide prior to our final ICBP protocol (Fig. 4) [28].

### Measures

We collected data about patient demographics, primary team, indications for procedure, and average weekly hospital occupancy. Occupancy is a potential confounder because a busier hospital may impact the intervention and outcomes such as shortening length of stay due to incentive to discharge patients quickly. We measured the effect of the intervention by comparing the number of adequate bowel preparations before and after the intervention. An adequate ICBP was defined as a total Boston Bowel Preparation Score (BBPS) ≥ 6 and all segment scores ≥ 2 or procedure description equivalent to “adequate” when score not provided.

Within the inpatient setting, a case may be delayed due to several factors unrelated to ICBP, such as acute non-GI related decompensation, dialysis, procedures, and patient preferences. Also, our intervention included improving nurse and patient recognition of readiness for procedure, which may increase delays in procedure. This delay would be preferable to having a timely procedure that is unsuccessful. Therefore, the primary outcome was percentage of “good ICBP”. “Good ICBP” was defined as adequate ICBP at initial colonoscopy regardless if procedure was delayed to the next day. The secondary outcomes were percent of “ideal ICBP”, which was defined as adequate ICBP and colonoscopy on the day as originally planned, and the LOS. LOS was defined as the date of discharge minus the date of first bowel preparation dose administration to eliminate unrelated pre-colonoscopy hospitalization time.

### Study of intervention & analysis

Demographic characteristics of patients and hospital crowdedness pre- and post-intervention were compared using t-tests and Wilcoxon sum rank tests for continuous variables, and chi-square and Fisher’s exact tests for categorical variables. We created a run chart to evaluate the process over time. A run chart is a health system engineering approach to evaluating the performance and changes in a process [29]. We were looking in the post-intervention period to see points above the baseline and less variation.

Generalized linear models estimated changes in outcomes before and after the intervention. Gaussian distribution with logarithmic link was used for LOS and binomial distribution with logit link was used for good and ideal preparation; these estimated percent changes in LOS and odds of good and ideal preparation, respectively. The models used linear splines for time to estimate [1] pre-intervention trend: the change in average outcome per week during the pre-intervention period; [2] the immediate effect of the intervention, reported as the change comparing the first week of the post-intervention period with the last week of the pre-intervention period; and [3] post-intervention trend: the sustained effect of the intervention reported as the change in the outcomes per week during the post-intervention period. The models included patient-specific robust variance estimates [30] to account for within-patient correlation due to repeated bowel preparation episodes in the same patient. If no statistically significant change per week (time trend) was observed in either pre- or post-intervention periods, the model was reduced to using one indicator variable for intervention as the primary predictor (coded as 0 for pre- and 1 for post-intervention period), without allowing for the time trends. This base model was extended to include additional adjustment for variables that differed between intervention and control periods. All tests were conducted at 0.05 level of statistical significance. The statistical analysis was performed using STATA version 15 software program [31].

## Results

### Phase 1

**Fig. 1:**
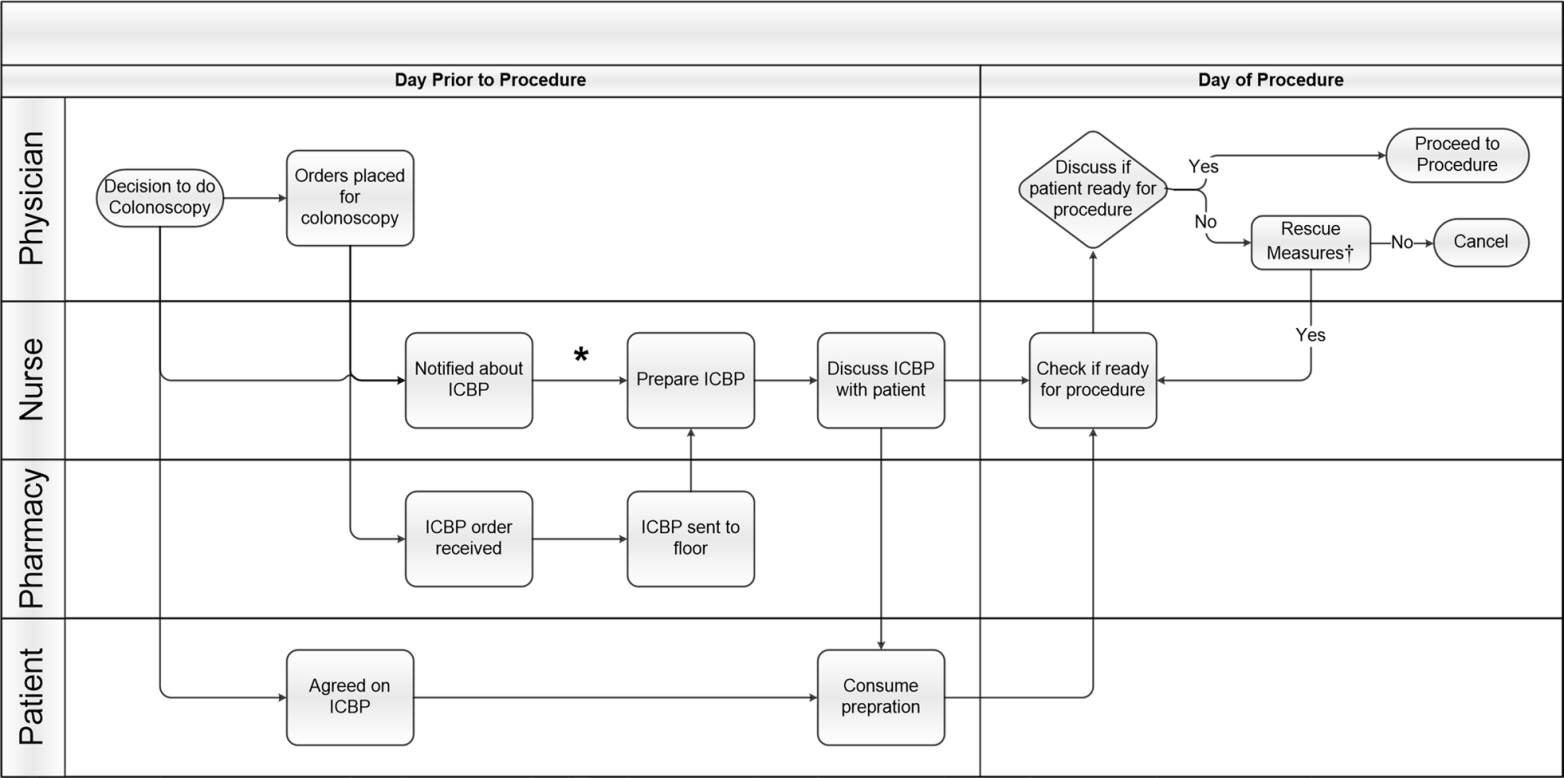
Inpatient Colonoscopy Bowel Preparation Process Map. Steps are shown at each level of care between physician, nurse, pharmacy, and patient from decision to do colonoscopy to the procedure. *Abbreviations: Inpatient Colonoscopy Bowel Preparation (ICBP).* *Units that perform ICBP frequently have ICBP stored in supply room on their unit. †Rescue measures: suppositories, enemas, additional bowel preparation

The tracer and process map (Fig. 1) identified key steps and decision points in the old ICBP process. The process started with the initial decision to scope made by the GI team which were comprised of fellows and attendings and were the same mix of experience levels throughout our entire study. Next, the primary team (residents, attendings, and advanced practitioners)placed orders, not consultants. Several steps performed by the patient, physicians, nurses, and pharmacists are performed on the day prior to the procedure and the morning of the procedure.

In the cause-effect diagram (Additional file 1: Fig. 1), causes for a poor ICBP based on our old process were placed into 6 categories: patient, supply, order, timing, provider, and communication. For example, we determined that orders for procedures were being placed late in the day due to late communication from GI to primary team of the procedure. Therefore, the patient was drinking the bowel preparation late into the evening. Another problem identified was patient education about the ICBP process was based solely on verbal communication as opposed to having supplemental visual aids. We found high variability in bowel preparation recommendations with the predominant approach being a single dose of 4 L the night before procedure. We determined that palatability was a problem because juices and powders used to improve the flavor of the solution were used infrequently because they were difficult to obtain by nurses. Lastly, we uncovered there was no standardization in the use of nasogastric tubes and in the time of day inaptient colonoscopies were scheduled.

### Phase 2

During the study phase of each PDSA cycle, we performed additional tracers and iterative discussions with patients and front-line providers at various points. For example, we engaged with the front line to discuss when an ICBP was performed on a floor where ICBPs are infrequently performed. These PDSA cycles allowed us to address key aspects of ICBP that were previously unspecified, such as nursing and patient concerns, tolerance of nasogastric tubes, and anti-emetic use. We developed several solutions described in detail below that contributed to our final protocol.

Early verbal communication by 4 p.m. of decision to scope to the primary team allowed time for pharmacy to deliver preparation medications and for nursing to prepare the medication. Since prior studies have demonstrated inpatients have a higher incidence of poor ICBP [2, 5, 6, 10, 12, 14] and guidelines recommend 6L bowel preparation for acute lower GI bleeding [32], our protocol included a 6L split-dose regimen with polyethylene glycol-electrolyte solution (PEG-ELS) with dose adjustment as needed. Based on recent publications, the use of polyethylene glycol (PEG) 3350 was phased out of practice at our institution and a sulfur-free version was added to formulary [18, 32, 33]. The ICBP start time for the first dose of 4L was between 4 p.m. and 6 p.m. the night prior to procedure at a rate of 1L per hour which allowed the patient to sleep. The second dose of 2L started at 5 a.m. and completed by 7 a.m.. Nurses used toilet specimen collectors to accurately assess stool for adequacy of ICBP. Enemas and suppositories were previously used as “rescue” in the morning were phased out of practice as they did not reach the right colon. Following evidence-based anesthesia recommendations [1, 34, 35], the inpatient colonoscopies were scheduled for a start time at 10 a.m. which allowed 2–3 h after completion of the morning dose.

We addressed communication between GI and primary team using a simple IT solution. Electronic Health Record (EHR) note templates were placed prominently in the recommendations section of the consultation note to streamline clear communication to the primary team and nursing. The templates included primary team and nursing instructions, recommendations for anti-emetics, steps to improve palatability, and the use of nasogastric tubes, where appropriate. Since the use of nasogastric tubes is recommended per guidelines for high volume bowel preparation [32], their use at the discretion of the GI and primary team was determined a priori.

**Fig. 2:**
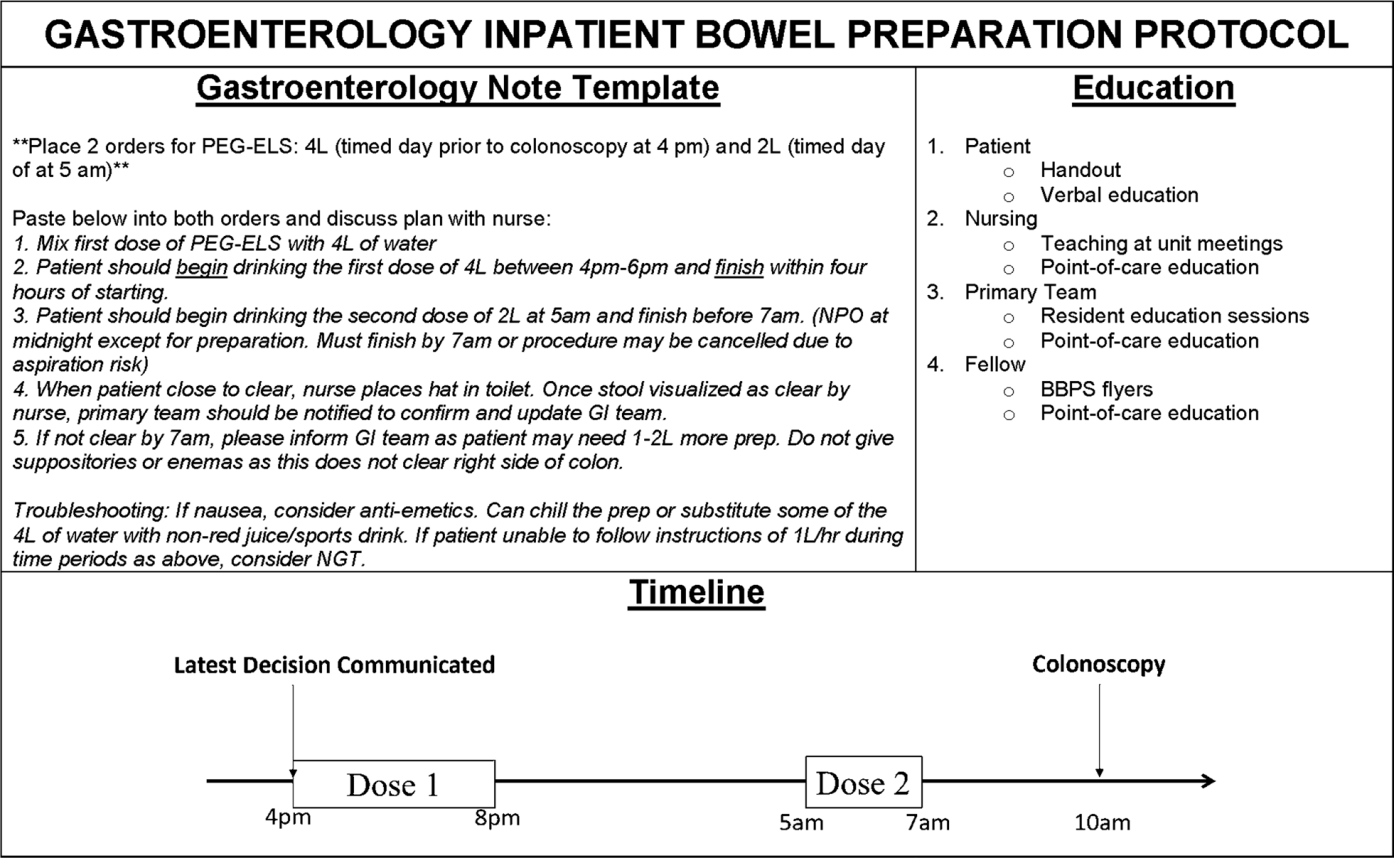
Gastroenterology Inpatient Bowel Preparation Protocol. GI: gastroenterology; BBPS: Boston Bowel Preparation Scale, PEG-ELS: polyethylene glycol-electrolyte solution

ICBP education was provided to patients, nurses, and physicians in the form of information sessions, point-of-care training, and handouts. Using the Pareto Principle, an engineering concept that a small percentage of participants constitute the majority of the action of interest, we identified the few nursing units and primary teams performing the most ICBP. We initially targeted these units and teams with education sessions, and these sessions were later expanded to include most other residents and nursing units in the hospital. Our patient education handout was described the importance a good ICBP, pictures, and frequently asked questions (Additional file 2: Fig. 2) [36]. Figure 2 shows the final protocol which includes an example of templated text in the electronic health record.

There were 120 and 129 bowel preparation episodes in 119 and 128 patients in the pre- and post-intervention groups, respectively. Table 1 shows the patient characteristics in the pre- and post-intervention periods. The individual factors of age, sex, race, ethnicity, primary team of internal medicine, and indications for procedure were similar between groups. The most common indication was bleeding. The system factor of hourly bed occupancy was a measure of how busy the hospital was in the week of ICBP and was statistically significantly higher in the post-intervention group. Therefore, we adjusted for this in the regression model.

**Table 1:** Characteristics of patients pre- and post-intervention to improve inpatient colonoscopy bowel preparation

	Pre-intervention	Post-intervention	*p* Value
	(n = 120)	(n = 129)	
Individual factors			
Age, mean (sd)	58.8 (17.34)	57.8 (17.5)	0.65
Male	61 (50.8)	63 (48.8)	0.75
Race, non-white	58 (48.3)	76 (58.9)	0.10
Primary team medicine service	113 (94.2)	113 (87.6)	0.07
Indication			0.85
Diarrhea, colitis	20 (16.7)	25 (19.4)	
Fecal transplant	1 (0.8)	3 (2.3)	
Abdominal pain	2 (1.7)	4 (3.1)	
Anemia	16 (13.3)	18 (14.0)	
Colon cancer	14 (11.7)	14 (10.9)	
Constipation	2 (1.7)	0	
Gastrointestinal bleeding	55 (45.8)	54 (41.9)	
Inflammatory bowel disease	10 (8.3)	11 (8.5)	
Systems factors			
Hourly inpatient occupancy, mean (range)	86.75 (85.61, 87.97)	90.32 (88.88, 90.88)	< 0.001

Data are presented as Number (%) unless otherwise noted

Sd: standard deviation

**Fig. 3:**
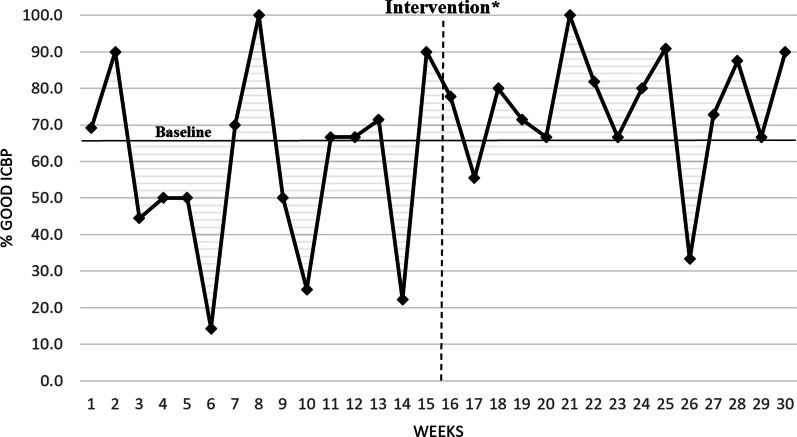
Run chart of percent good inpatient colonoscopy bowel preparations with pre-Intervention baseline to compare with post-intervention. ICBP: Inpatient Colonoscopy Bowel Preparation. *Represents time period for development and deployment of intervention between pre and post-intervention periods

**Fig. 4:**
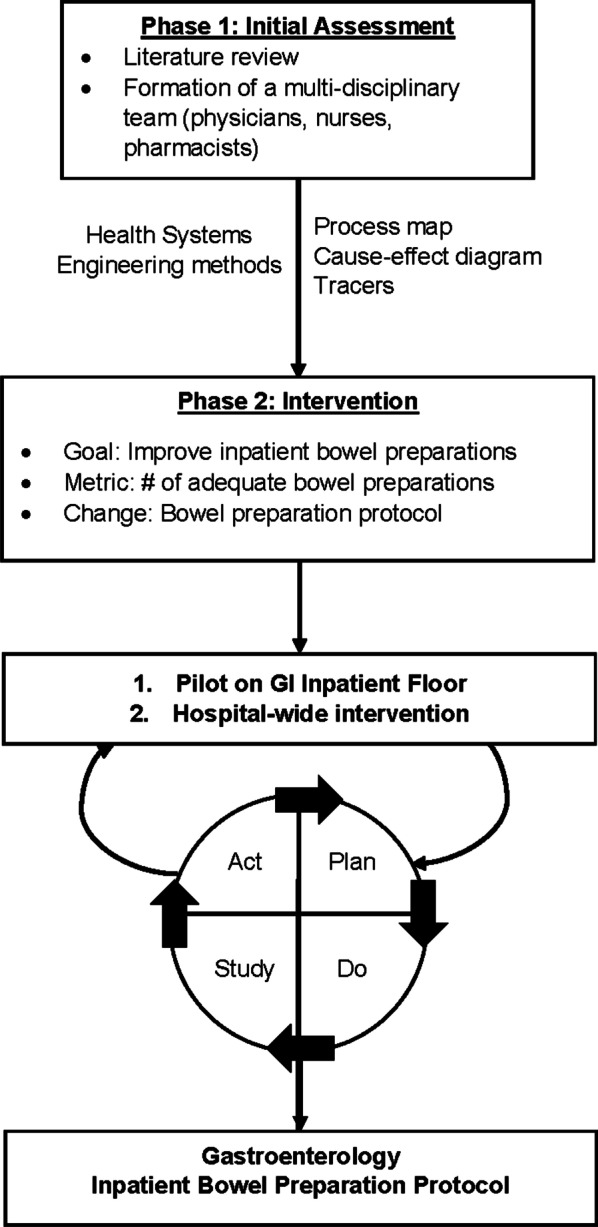
Inpatient Bowel Preparation Quality Improvement Methodology. Outline of our methods using Health Systems Engineering principles and the Model for Improvement to improve inpatient bowel preparations. GI: gastroenterology

We calculated the percentage of planned colonoscopies per week that resulted in a good ICBP for the pre-intervention period. The median percentage of good ICBP for the pre-intervention period was identified as our baseline which was 66.7%. We used this baseline to compare our post-intervention period. Each week, we evaluated our intervention through PDSA cycles using the new data as a guide. Figures 3, 4 shows a run chart of the percentage of good ICBP by week for the pre- and post-intervention periods. A “shift”, 6 or more consecutive points above the baseline [29], is seen from week 18 to week 25. One-point deviations, such as the one seen in week 26, are considered noise in the process due to the stochastic nature hospital processes, so the focus is on trends. Looking overall at this run chart, one can appreciate the improvement in good ICBP and increased consistency in the post-intervention period.

Prior to intervention there was no statistically significant time trend (the percentage of adequate ICBP was not increasing or decreasing) which demonstrated the intervention was not following an already upward trajectory of improving ICBP. Similarly, we did not observe statistically significant time trend in the post-intervention period which demonstrates stability of the impact of the intervention over time. For example, if we had found a downward trend in ICBP in the post-intervention period, we would be concerned the intervention effect was not sustained. Thus, below we report the results of the models with intervention as the primary predictor to evaluate the impact of the intervention. In addition, since the pre and post periods had different occupancy levels, this variable was added to the model for adjustment.

Our regression model showed the pre-intervention percent of good ICBP was 61% compared to 74% after the intervention with estimated odds ratio of a good ICBP comparing post- to pre-intervention of 1.87 (95% CI 1.09–3.21, *p *value  = 0.02). The estimated odds ratio did not substantially change after adjustment for average weekly occupancy (Table 2).

**Table 2:** Estimates from generalized linear model for adequate bowel preparations and length of stay before and after the intervention in unadjusted and adjusted models

Study outcome	Pre vs. post	Unadjusted model	Adjusted model
		Relative risk (post / pre) [95% confidence interval]	Relative risk (post / pre) [95% confidence interval]
Good^†^ ICBP	60.8% vs. 74.4%	1.873*	1.947*
		[1.093,3.211]	[1.025,3.697]
Ideal^‡^ ICBP	53.3% vs. 69.0%	1.947*	1.901*
		[1.160,3.266]	[1.024,3.531]

		Ratio of means (post / pre) [95% confidence interval]	Ratio of means (post / pre) [95% confidence interval]
Mean length of stay, days	8 vs. 6	0.759	0.852
		[0.538,1.069]	[0.617,1.179]

Adjusted model includes average weekly occupancy. ICBP: inpatient colonoscopy bowel preparations.

^†^Good: colonoscopy delayed and adequate bowel preparation when performed.

^‡^Ideal: colonoscopy not delayed and adequate bowel preparation when performed. *p < *0.05*

The regression analysis showed that before the intervention the percent of ideal ICBPs was 53% compared to 69% after the intervention. The estimated odds ratio of ideal ICBP comparing post- to pre-intervention was 1.95 (95% CI 1.16–3.27, *p *value  = 0.01). The estimated odds ratio of ideal ICBP did not substantially change after adjustment for average weekly occupancy. There was estimated 25% decrease in LOS in the post intervention compared to pre-intervention period, from mean of 8 days in the pre-intervention period to mean of 6 days in the post-intervention period. This decline, however, did not reach statistical significance (95% CI 0.54–1.07, *p *value  = 0.1).

## Discussion

### Summary

This study demonstrated that HSE principles and a multi-disciplinary team can be used to identify problems in the ICBP process and to implement solutions such as multi-faceted education and IT interventions to significantly improve the number of adequate ICBP. A strength of this study was the systematic problem analysis and standardization through a comprehensive protocol. Additionally, the ICBP intervention immediately improved adequate ICBP which was sustained for 15 weeks in the post-intervention period.

This intervention decreased mean LOS by 25%. Although this failed to reach statistical significance, during the study period our institution had an overall increase in LOS. Thus, decreasing LOS by 25% was important clinically and small improvements in LOS can have substantial impact on hospital throughput. However, it is difficult to interpret the change in estimates in unadjusted versus adjusted models since the relationship between occupancy and LOS is difficult to untangle. Many factors interplay that could lead to increase in one leading to an increase or decrease in the other. Our study shows that after controlling for occupancy this intervention impacts LOS, but more work is needed to determine strategies to have a larger effect.

### Interpretation & lessons learned

The comprehensive list of identified problems and solutions to the ICBP process are outlined in Table 3. These are likely common in most institutions, so this table can serve as an initial guide for others. Early, effective communication about the decision to scope is critical. Early morning ICBP consumption between 12 a.m. to 5 a.m. generally is to be avoided when the patient is likely more prone to falling. This study highlights the many stakeholders, including patients, involved in the ICBP process. Therefore, educating everyone about their role in the ICBP process and the importance of an adequate bowel preparation (i.e. diagnosis, treatment, and adverse events or repeat procedures) is paramount.

**Table 3:** Potential problems and solutions at patient, nurse, physician and system level for the bowel preparation process for inpatient colonoscopy

Level	Potential problems	Potential solutions
Patient	Poor palatability	Switch to Polyethylene glycol-electrolyte solution with flavor packets of 4 flavors attached to bottle and no sulfur taste
		Chill bowel preparation
		Mix with flavor powders or non-red juice
	Unable to drink fast enough due to volume or nausea (assuming obstruction not suspected) or altered mental status (i.e. delirium or dementia)	Anti-emetics
		Nasogastric Tube
	Not following instructions	Patient education handout
		Family involvement
	Inpatient status ± underlying risk factors for poor bowel preparation	Consider 6-L bowel preparation
		Consider 2-day bowel preparation
	Flushes bowel movement before nurse evaluating	Nursing places toilet hat when close to ready
Nursing	Floor nurse protocol cannot require bedside checks more frequently than every 4 h	Encourage family to help
		Recruit medical assistant participation
		Use of technology for reminders
	Unclear importance of bowel preparation and instructions highly variable	Standardize instructions
		Nursing education sessions
		Endoscopy and floor nurses discuss day prior to procedure
	Original nurse communication with instructions acknowledged by day shift nurse and not viewed by night shift nurse	Instructions in medication order so viewable when administering
		Orderset with timed instructions
	Variable reporting of readiness for procedure	Nursing education and picture of readiness on patient education
		Toilet hat
		Endoscopy and floor nurses discuss morning of procedure
Physician	Preparation recommended by gastroenterology highly variable leading to confusion	Create protocol for standardization
	Instructions from gastroenterology not clear and/or written in notes	Electronic note templates for easy use in gastroenterology notes
	Due to nature of complex inpatient consult service, decision-to-scope communicated late (i.e., after 6 pm) to primary team	Set mutually agreed upon expectation for early communication by gastroenterology with a set latest time (i.e., 4 pm)
	Ordering suppositories and enemas as “rescue” in the morning leads to false sense patient is clear when right side of colon is not	Using more bowel preparation instead of suppositories and enemas
		Conversion to 2-day preparation
	Boston Bowel Preparation Score not properly documented. This could be knowledge gap or due to busy inpatient consult service while scoping. Procedure notes written at end of day leading to memory and bias	Scoring education
		Document score in Brief-Op note immediately post-procedure for reference later when writing procedure note
	Primary team orders differently than gastroenterology recommendations	Primary team education
		Orderset
System	Amount of bowel preparation consumed not documented	Fellow or nurse go to bedside to document amount drank
		Educate nursing day prior to document in medical record
	Lag time between order and administration	Stock bowel preparation in pyxis on specific floors
	Long chain of communication: GI, primary team, day nurse, night nurse	Set protocol for communication expectations, note templates, ordersets
	Dietary keeps flavor mix packs and nursing unable to get after certain hour	Stock flavor packs on floor or use Polyethylene glycol-electrolyte solution with flavor packets of 4 flavors attached to bottle

Standardizing our protocol to 6 L was simply one aspect of our multi-pronged approach and was adjusted on an individual basis based on clinical judgement. Our success with 6 L is aligned with the literature [18] that also suggests better outcomes with high volume for inpatients. Randomized controlled trials are needed to validate the populations that would derive maximal benefit from this approach.

### Limitations

As with any QI study there are some methodologic limitations. Firstly, this is a single-center QI study conducted at a tertiary medical institution with a high-volume inpatient colonoscopy case load. Therefore, the generalizability may be limited (e.g., secondary hospitals), so future studies at multiple centers with varying patient populations would be useful. Notably, the sample size was reasonably large with 249 ICBP episodes; however, a larger sample may have led to a larger effect size for LOS. Although we cannot make inference about which specific part of our new ICBP protocol led to the greatest improvement in ICBP, we have shown that a comprehensive approach is possible despite the difficulty of coordinating many moving parts. Secondly, this QI project occurred over the course of an academic year. Consequently, there may have been sampling bias, and we were not able to control for increased experience from new residents and fellows partially contributing to improved ICBP. For example, there may have been variation in the scoring of the BBPS as this was not explicitly calibrated in this study; however, all fellows were trained similarly on the BBPS, so their scoring was likely standardized across people and time. The sustainability and significant improvements in ICBP seen suggest that the experience of trainees would not negate these findings. Thirdly, this study did not control for some elements that are known to increase risk of a poor bowel preparation (i.e. certain comorbidities, past failed bowel preparations, and narcotics) due to limited data availability, so this could have led to an increased risk of bias. Future research may prospectively address comorbidities and the effects of this protocol on patient satisfaction, quality of life, compliance, and adequacy of ICBP.

## Conclusion

This study highlights how HSE techniques when combined with a multi-disciplinary team, multi-faceted education, and information technology interventions can be used to improve complex hospital processes such as ICBP to improve overall patient care and important hospital metrics such as LOS.

The sustainability of QI interventions is important to provide long-term quality care. We showed an immediate improvement that was sustained over the entire 15 weeks post-intervention. The protocol changed fundamental systems components of the ICBP process that will enable long-term culture change. Furthermore, this QI intervention paves the way for implementation of an electronic order set version of the protocol. Building on interventions centered on education with IT solutions can help to sustain the project’s positive effects.

To sustain an effective ICBP protocol, we continued to reinforce this change in culture around ICBP by performing education sessions after our intervention period. Education sessions with nursing and primary teams emphasized the importance of a good ICBP, the timing of the ICBP, and common pitfalls. Meaningful time points for education are either monthly meetings with residents or nurses and at transition points in training (i.e. new trainees in July). Other important sustainability components are periodic project updates for stakeholders and identifying units that are top performers to be champions and motivate other units. Following this intervention, the patient handout was made viewable on our institution’s patient education website and printable directly from the EHR which increased compliance.

Utilizing HSE approaches have been generalized to improve the care of patients in various medical areas [24–26], and we have presented ICBP is another example. We have been forthcoming and transparent about our techniques and process, so our colleagues in GI and other procedural fields have an initial framework to apply locally and adapt to their specific problematic processes. By addressing issues at patient, provider, and system levels with health systems engineering principles, patient safety and quality care can be meaningfully improved.

## Supplementary Information

**Additional file 1.** **Figure 1**. Cause-Effect Diagram of possible reasons for a poor bowel preparation. BP: Bowel Preparation, PCT: Patient care technician, PEG: Polyethylene Glycol

**Additional file 2.** **Figure 2**. Patient education sheet for preparing for colonoscopy.

## Data Availability

The datasets during and/or analyzed during the current study available from the corresponding author on reasonable request.

## Abbreviations

ICBP: Inpatient colonoscopy bowel preparation
LOS: Length of stay
HSE: Health systems engineering
QI: Quality improvement
GI: Gastroenterology
PDSA: Plan-do-study-act
BBPS: Boston bowel preparation score
PEG-ELS: Polyethylene glycol-electrolyte solution
PEG: Polyethylene glycol
HER: Electronic health record
