# Supplementary figures and images for: A patient-centered framework for health systems engineering in gastroenterology: improving inpatient colonoscopy bowel preparation
Source: BMC Gastroenterol. 2021 Feb 27;21:89. doi: 10.1186/s12876-021-01661-4 (PMC7912514; doi:10.1186/s12876-021-01661-4)

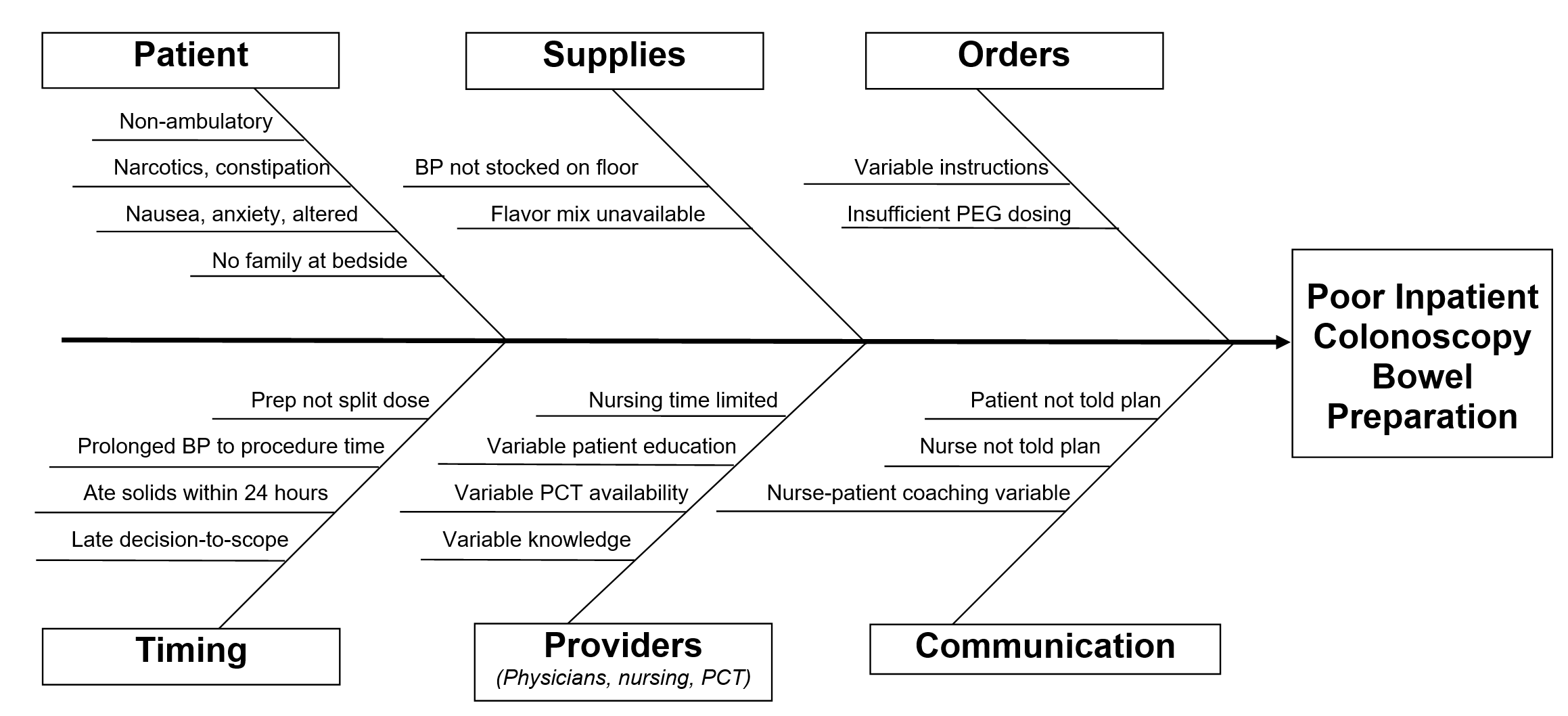

Supplement: Supplementary file 1 — Additional file 1. Figure 1. Cause-Effect Diagram of possible reasons for a poor bowel preparation. BP: Bowel Preparation, PCT: Patient care technician, PEG: Polyethylene Glycol [file 12876_2021_1661_MOESM1_ESM.tif]
