# Supplementary material for: A patient-centered framework for health systems engineering in gastroenterology: improving inpatient colonoscopy bowel preparation
Source: BMC Gastroenterol. 2021 Feb 27;21:89. doi: 10.1186/s12876-021-01661-4 (PMC7912514; doi:10.1186/s12876-021-01661-4)
Supplement: Supplementary file 2 — Additional file 2. Figure 2. Patient education sheet for preparing for colonoscopy. [file 12876_2021_1661_MOESM2_ESM.pdf]

Preparing for Colonoscopy

What is a colonoscopy?
Colonoscopy is a test to view the inside of your lower digestive tract (colon and rectum). Sometimes it can also show the last part of the small intestine (ileum). During the test, small pieces of tissue may be removed for testing. This is called a biopsy. Small growths, such as polyps, may also be removed. For a successful colonoscopy, your colon needs to be very clean to help your doctor see the colon and any polyps, tumors, bleeding, or anything else abnormal in your colon.

Why is the prep important?
The goal is to have your digestive tract clear of all fecal material, food and anything else that may be present to allow your care team to conduct a complete examination. If you have not successfully completed your bowel preparation, your exam may be postponed or rescheduled.

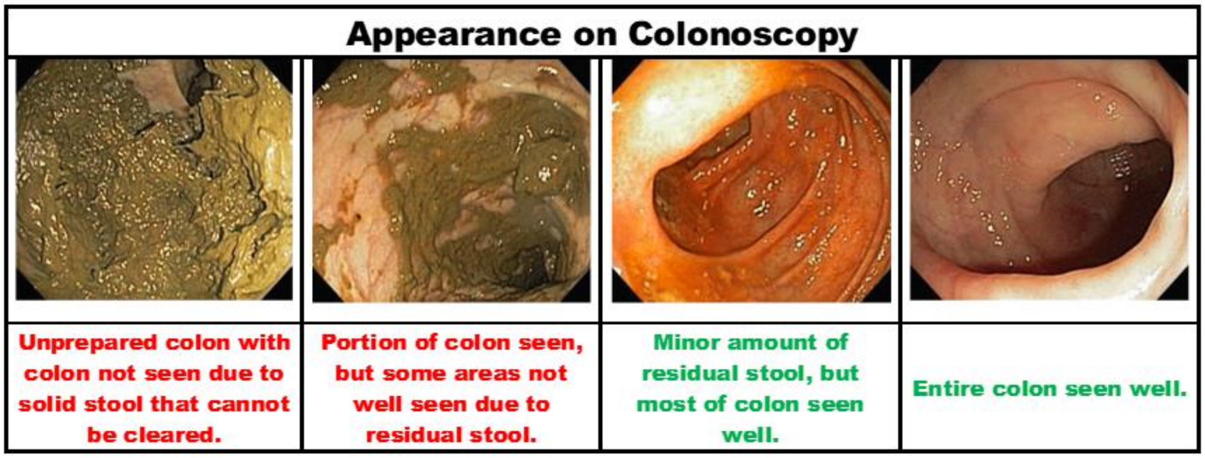

How do I prepare for a successful colonoscopy?
Your doctor will order you a clear liquid diet the day before your procedure. Please do not eat any food or drinks that are red in color (e.g., cranberry juice) because it may look like you are bleeding during the examination. You will be given a preparation. It is important to drink it as recommended below.

- The evening before your procedure, you will be given a preparation to drink as prescribed.
- Drink an 8 oz. glass of preparation every 10-15 minutes until the amount is complete. This will take approximately 2-4 hours.
- The morning of your procedure, you will be given a half-gallon of preparation to drink. Drink an 8 oz. glass of preparation every 10-15 minutes until the entire half-gallon is complete. This will take approximately 2 hours.
- You must stop drinking all liquids 3 hours before the colonoscopy for a safe procedure.

It is important to:
- Tell your nurse when your bowel movement is clear yellow.
- Use the toilet hat to catch the bowel movement for the nurse to see.
- Do not flush the toilet.

Tips for completing the prep:

- To improve the taste of preparation, it can be chilled prior to giving it to you and you may drink it through a straw.
- Mix preparation with flavor packs
- You can ask for a bedside toilet if you have difficult walking to bathroom frequently.
- You may need to drink the prep while on the toilet to complete it.
- Tell your nurse if you develop abdominal pain, bloating, or nausea while drinking the medication.
- Watery bowel movements will continue for several hours after completing the bowel prep.
- Be sure to keep drinking water, so you do not become dehydrated from the prep.
- If you have trouble drinking the prep, you may need to have a tube put in through your nose that goes to your stomach to help you complete the prep.

Results:

- Your bowel movements will lighten in color as time goes on.
- You will need to look in the toilet after you have a bowel movement to see the color.
- There should not be any solid matter.

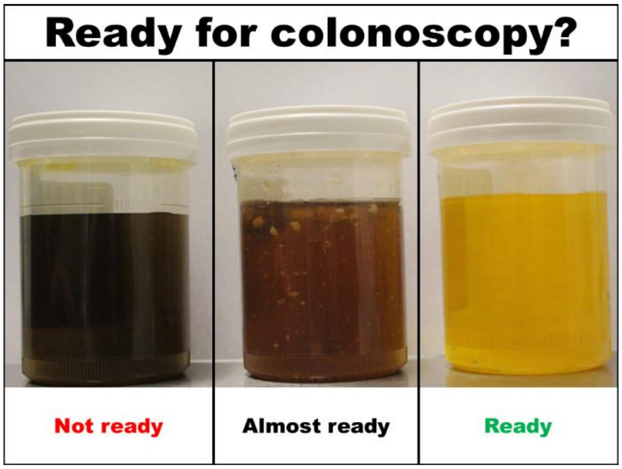

Reference:

Lai EJ, Calderwood AH, Doros G, Fix OK, Jacobson BC. The Boston Bowel Preparation Scale: A valid and reliable instrument for colonoscopy-oriented research. *Gastrointestinal endoscopy*. 2009;69(3 Pt 2):620-625. doi:10.1016/j.gie.2008.05.057.
